# Health Emergency and Disaster Risk Management During the COVID-19 Pandemic: A Case Study from Italy: Emanuela Parotto

**DOI:** 10.1093/eurpub/ckac131.056

**Published:** 2022-10-25

**Authors:** A Lamberti-Castronuovo, E Parotto, L Ragazzoni, M Valente

**Affiliations:** 1 CRIMEDIM, Università del Piemonte Orientale, Novara, Italy; 2 Dipartimento di Chirurgia DIDAS, Azienda Ospedale Università Padova, Padua, Italy

## Abstract

**Background:**

The COVID-19 pandemic has profoundly impacted societies, influencing countries’ Health Emergency and Disaster Risk Management (H-EDRM) systems. By taking Italy as a case study, this research aimed to investigate the response to the pandemic focusing on challenges, response strategies, lessons learned and implications for H-EDRM, with an emphasis on health workforce, health services delivery and logistics.

**Methods:**

This was a retrospective observational study using qualitative methodology. Data was collected via semi-structured interviews and analyzed according to the H-EDRM framework. Multiple interviewees were selected to obtain a holistic perspective on the Italian pandemic response. Stakeholders from five sectors (policymaking, hospital, primary care, third sector, lay community) from three of the most impacted Italian regions (Piemonte, Lombardia, Veneto) were interviewed, reaching 15 interviewees in total.

**Results:**

With regard to human resources, the main themes concerned the shortage of personnel, inadequate training, poor occupational health, and lack of multidisciplinarity. Regarding health services delivery, interviewees reported weakness of public health, hospital, and primary care systems. With regard to logistics, the following themes emerged: inadequate infrastructures, shortage of supplies, issues with transportation systems, and weak communication channels. Lessons learned stressed the importance of considering pragmatic disaster preparedness and the need for cultural and structural reforms.

**Conclusions:**

Implications that emerged from this study can inform advancements in disaster management in Italy.

Acknowledgments: This study was conducted in collaboration with the Department of Public Health and Health Policy, Hiroshima University, Japan and funded by the World Health Organization Kobe Centre for Health Development (WKC-HEDRM-K21001).

*ALC and EP are both first authors

**Key messages:**

• Findings show that great interconnection of sectors is key in overcoming challenges and for future disaster preparedness.

• Lessons learned contribute to translating the H-EDRM precepts into practice.

